# Matrix Effect Study and Immunoassay Detection Using Electrolyte-Gated Graphene Biosensor

**DOI:** 10.3390/mi9040142

**Published:** 2018-03-23

**Authors:** Jianbo Sun, Yuxin Liu

**Affiliations:** Lane Department of Computer Science and Electrical Engineering, West Virginia University, Morgantown, WV 26506, USA; jnsun@mix.wvu.edu

**Keywords:** graphene, electrolyte-gated field effect transistor, matrix effect, immunoassay

## Abstract

Significant progress has been made on the development of electrolyte-gated graphene field effect transistor (EGGFET) biosensors over the last decade, yet they are still in the stage of proof-of-concept. In this work, we studied the electrolyte matrix effects, including its composition, pH and ionic strength, and demonstrate that variations in electrolyte matrices have a significant impact on the Fermi level of the graphene channel and the sensitivity of the EGGFET biosensors. This is attributed to the polarization-induced interaction between the electrolyte and the graphene at the interface which can lead to considerable modulation of the Fermi level of the graphene channel. As a result, the response of the EGGFET biosensors is susceptible to the matrix effect which might lead to high uncertainty or even false results. Then, an EGGFET immunoassay is presented which aims to allow good regulation of the matrix effect. The multichannel design allows in-situ calibration with negative control, as well as statistical validation of the measurement results. Its performance is demonstrated by the detection of human immunoglobulin G (IgG) from serum. The detection range is estimated to be around 2–50 nM with a coefficient of variation (CV) of less than 20% and the recovery rate for IgG detection is around 85–95%. Compared with traditional immunoassay techniques, the EGGFET immunoassay is label-free and ready to be integrated with microfluidics sensor platforms, suggesting its great prospect for point-of-care applications.

## 1. Introduction

Field effect transistor (FET) biosensors are operated by measuring the conductance changes of a channel induced by the binding of target molecules to it. Compared with other biosensing techniques, FET biosensors are particularly favored for their potential in portable and point-of-care biomedical applications due to their high sensitivity, simple configuration and cost-effective mass producibility [1,2]. Over the past decades, the development of FET biosensors has been significantly boosted by the introduction of nanomaterials, such as silicon nanowire (SiNW), carbon nanotube (CNT) and graphene [3]. Among them, the single-atom-layer structure and unique properties of graphene, including its superior electronic characteristics, facile functionalization and good biocompatibility, make it an attractive candidate as a building block of FET biosensors [2,4,5].

Significant progress has been made in the development of electrolyte-gated graphene field effect transistor (EGGFET) biosensors over the last decade [2]. Various targeting analytes have been demonstrated for detection, including nucleic acids [6], proteins [7], metabolites [8] and other biologically relevant analytes [9]. EGGFET biosensors have shown sensitivities as low as attomolar and high selectivity towards target biomolecules [10]. EGGFET biosensors have also been applied for electrophysiological measurements due to their high spatial resolution and low noise level, such as the detection of electrical activity of electrogenic cells [11,12]. Efforts have been made to overcome the Debye screening effect which is one of the main factors that limits the sensitivity of EGGFET biosensors [13,14,15]. Other strategies have also been applied to further improve their performance, such as gold nanoparticle decoration [16]. In addition, the rapid development of the preparation techniques of graphene also contributes to the maturation of EGGFET biosensors and it outperforms SiNW and CNT in performance and mass producibility [17,18].

Despite the reports of outstanding performances, most results have been based on experiments using simplified samples in the laboratory setting [1,2]. In EGGFET biosensors, the graphene channels are directly exposed to the electrolytes, which might be variant in composition, concentration and pH, etc. These variables might originate from the sample themselves and the sample handling processes, which might affect the operation of the EGGFET biosensors. For example, the pH of human blood is normally regulated between 7.35 and 7.45, but many conditions and diseases can interfere with pH control and cause the blood pH to fall outside of healthy limits, such as acidosis and alkalosis [19]. During actual measurement, samples are often diluted to make the analyte level fall within the detection range of the biosensors, which may also change the electrolyte matrix composition. Furthermore, in EGGFET biosensors, the gate voltage is applied to the electrolytes, and the electrical double layer (EDL) at the electrolyte–graphene interface serves as the gate dielectric; therefore, it is of critical importance to study if and how variance in the electrolytes affects the operation of the EGGFET biosensors. Until now, detailed understanding of the electrolyte–graphene interaction and its impact on the electronic transport in graphene is still lacking [1], despite being critical for the design and operation of the EGGFET biosensors, especially for their practical applications.

In this work, we studied the impact of the variance in electrolyte matrices on EGGFET biosensors—known as the matrix effect—by varying the composition, ionic strength and pH of the electrolytes. The underlying mechanisms were discussed with regarding to the strong polarization-induced interaction between the electrolytes and the graphene and its impact on the Fermi level of the graphene channel. The influence of the matrix effect on the gate potential and the sensitivity of the EGGFET biosensors were also studied. For the regulation of the matrix effect, we present an immunoassay based on the EGGFET immunosensors. Its performance was demonstrated by the successful detection of human immunoglobulin G (IgG) from serum in spike-and-recovery experiments.

## 2. Experiments

### 2.1. Design of the EGGFET Immunoassay

**Figure 1:**
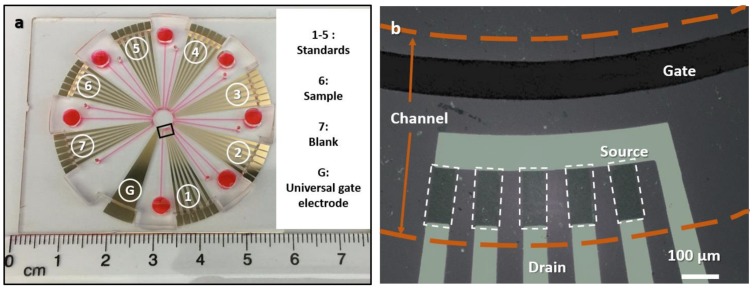
Electrolyte-gated graphene field effect transistor (EGGFET) immunoassay chip. (**a**) The prototype of the EGGFET immunoassay chip with the sample delivery channels filled with red food dye. The numbers indicate the seven immunosensor sets with their function assignment as specified on the right; (**b**) An enlarged view of the EGGFET immunosensor set that was fabricated on SiO_2_/Si substrate for the visualization of graphene (as indicated by the white dashed frames). The sample delivery channel is indicated by the brown dashed line.

A prototype of the EGGFET immunoassay chip and an enlarged view of the EGGFET immunosensor configuration are shown in Figure 1a,b, respectively. The chip consists of 7 immunosensor sets that are distributed in a circular form, including five sets (numbers 1–5) for calibration curve (standards) measurement, one (number 6) for sample measurement, and one (number 7) for negative control. A common ring-like Ag/AgCl pseudo-reference electrode works as the gate (G). Each immunosensor set consists of 5 EGGFET immunosensors, which allows for the statistical validation of the measurement results. The dimension of the graphene channels is 150 μm × 75 μm. The sample-delivery channels are filled with red food dye for demonstration.

### 2.2. Fabrication of the EGGFET Immunoassay Chip

#### 2.2.1. Graphene Transfer and Electrolytic Cleaning

Poly(methyl methacrylate) (PMMA, 950 kDa, 4% in anisole, MicroChem, Westborough, MA, USA) was spin-coated on CVD graphene (on copper, ordered from Graphene Supermarket, Calverton, NY, USA) at 2000 rpm for 30 s and then cured at 120 °C for 1 min. The sample was treated by oxygen plasma asher at 30 W for 90 s to remove the backside graphene, and then was placed to be floating on iron nitrate (Fe(NO_3_)_3_ (0.7 M, Sigma Aldrich, St. Louis, MO, USA) for 3 h to etch the copper. The PMMA/graphene film was lifted by a glass slide (cleaned by ultrasonication in acetone, isopropyl alcohol (IPA) and deionized (DI) water for 5 min, successively) and allowed to dry for 1 h at room temperature. The sample was then heated at 75 °C for 30 min to improve the contact between graphene and the substrate. The PMMA was removed by acetone and rinsed with IPA and DI water, respectively. The sample was annealed at 250 °C in a nitrogen atmosphere with a flow rate of 1000 sccm to remove the PMMA residues. To remove the post-annealing residues, the graphene was cleaned using an electrolytic method at −5 V vs. an Ag/AgCl reference electrode in 0.5 M sulfuric acid for 30 min as we reported previously [20].

#### 2.2.2. Fabrication of the Electrodes and the Graphene Channels

After washing with acetone and IPA and drying at 75 °C for 30 min, 5 nm nickel and 45 nm gold were deposited on the sample using e-beam evaporation. The electrodes were patterned by photolithography using AZ5214E photoresist (MicroChemicals, Ulm, Germany) followed by etching in gold etchant (Gold Etch TFA, Transene Company, Inc., Danvers, MA, USA) and nickel etchant (Nickel Etchant TFB, Transene) for 10 s, successively. A second photolithography process was applied to create a shielding photoresist layer on the graphene channel, and then the graphene channel was patterned by oxygen plasma etching (100 W for 90 s with oxygen flow at 49 sccm). The shielding photoresist layer was finally removed using acetone followed by rinsing with IPA and DI water.

A three-electrode cell with a standard Ag/AgCl reference electrode, a gold wire coil counter electrode, was used for the electroplating of Ag/AgCl on the as-fabricated gold electrode. The schematic and experimental setup are shown in Supplemental Section 1 and Figure S1. For the electroplating of silver, 0.3 M silver nitrate (AgNO_3_) and 1 M ammonia (NH_3_, aq) solution were injected into the chamber. First, an oxidative pre-treatment at +0.95 V was applied for 30 s. For better results, the sample was placed in a vacuum for 30 min to remove the dissolved oxygen and the microscopic gas bubbles on the electrode surface. Then the electroplating was driven at −0.5 mA for 300 s, resulting in a Ag layer of around 5 μm in thickness. A quantity of 0.1 M HCl solution was injected into the chamber after rinsing with DI water, and the chloridization was driven at +0.2 mA for 60 s. After removing the electroplating chamber, the sample was rinsed with DI water.

#### 2.2.3. Integration with the Microfluidics Channel

The sample delivery channel was made of polydimethylsiloxane (PDMS) using the soft lithography technique. Due to the vulnerability of graphene, conventional oxygen plasma cannot be used for the activation of the glass surface for bonding with PDMS. As an alternative, the EGGFET chip was immersed in 0.1 M NaOH solution for 30 s and then rinsed with DI water with a layer of DI water left on the EGGFET chip afterwards. The PDMS sample delivery channel was activated with oxygen plasma and then applied onto the EGGFET chip. The alignment of the sample delivery channel and the EGGFET chip was accomplished under a microscope. The interfacial water layer serves as the lubricant and prevents the immediate bonding of glass and PDMS, while preserving their bonding capability. The aligned sample was then placed in a 60 °C oven for 3 h for bonding.

#### 2.2.4. Functionalization of the Graphene Surface for IgG Detection

**Figure 2:**
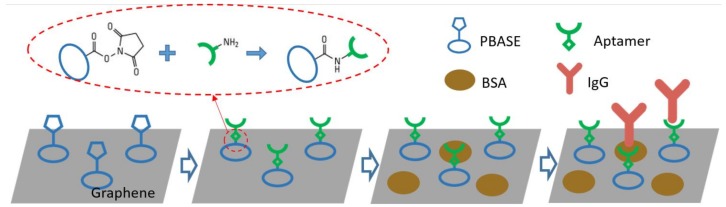
Schematic diagram for the functionalization of the graphene for immunoglobulin G (IgG) detection.

A schematic diagram in Figure 2 shows the functionalization process of the graphene surface for IgG detection. After rinsing the channel with dimethyl sulfoxide (DMSO, VWR), 1-pyrenebutyric acid *N*-hydroxysuccinimide ester (PBASE, Sigma Aldrich, 10 mM dissolved in DMSO) was injected into the channels and kept for 2 h. PBASE can be adsorbed on graphene through a π–π interaction without damaging its electrical properties, and it is widely used for the functionalization of graphene and carbon nanotubes [5]. A 5′-amino modified IgG aptamer (100 μM in 1× PBS, Base Pair Biotechnologies, Pearland, TX, USA) was then injected in the channels and incubated for 3 h to allow the conjugation with PBASE. The conjugation is achieved by the amide bonding between the reactive *N*-hydroxysuccinimide (NHS) ester in PBASE and the amine group on the 5′ end of the IgG aptamer. The remaining unconjugated sites were blocked by bovine serum albumin (BSA, 10% *w*/*v* in 1× PBS, Sigma Aldrich) after rinsing with 1× PBS. X-ray photoelectron spectroscopy (XPS) and Raman spectroscopy were performed to study the functionalization of the graphene surface. The transfer curves of the EGGFET biosensors were measured after each step of functionalization. Details are provided in the Supplemental Section 2 and Figure S2.

### 2.3. Electrical Measurement

The electrical measurements were conducted using Keithley 4200 SCS (Tektronix, Inc., Beaverton, OR, USA) and a Micromanipulator probe station. The operation parameters, including the gate voltage scan rate, gate voltage setting and drain-source voltage, were optimized to minimize the hysteresis-induced deviation and maximize the transconductance (Supplemental Section 3, Figure S3). The data were collected by measuring the five parallel channels in each immunosensor set, and the results were obtained through statistical analysis of the measured results. The open circuit potential (OCP) of the Ag/AgCl pseudo-reference electrode with respect to the standard Ag/AgCl reference electrode (CHI111, CH Instruments, Inc., Austin, TX, USA) was measured using a Gamry Interface 1000T potentiostat (Gamry Instruments, Warminster, PA, USA).

## 3. Results and Discussion

### 3.1. Operation Principles of the EGGFET Biosensors

**Figure 3:**
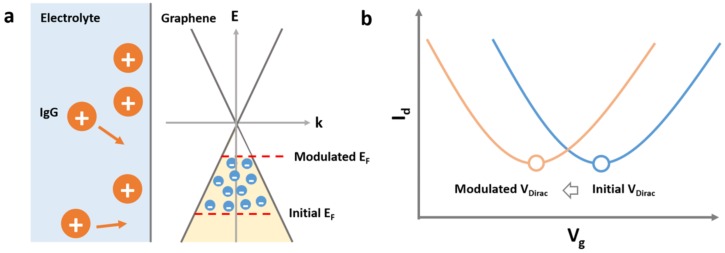
Operation principles of the EGGFET biosensors. (**a**) The schematic diagram shows the modulation of the Fermi level in graphene upon the binding of IgG molecules; (**b**) the schematic diagram shows a negative shift of the transfer curve upon the binding of IgG molecules.

The operation of the EGGFET biosensors is based on the modulation of the Fermi level in the graphene channel by electrostatic gating upon specific adsorption of the charged biomolecules. Graphene is a zero-bandgap semiconductor with its conduction band and valance band meeting at the Dirac points with linear energy dispersion [21]. At low energy levels, the Fermi level in graphene is sensitive to its carrier density, owning to the low density of states. As shown in Figure 3a, the specific binding of the positively charged molecules (i.e., IgG) can cause the accumulation of electrons in the graphene channel (n-doping) through the electrostatic gating effect and the corresponding positive shift in the Fermi level. The transfer curve measurement can be used to locate the Fermi level in graphene. Due to the unique band structure of graphene, EGGFET biosensors exhibit an ambipolar electric field effect, and the minimum conductivity is obtained when the Fermi level of graphene coincides with the Dirac point [22] (Figure 3b). The gate voltage with the minimum conductivity is typically used for parameterizing the Fermi level, which is typically referred to as the Dirac voltage ($V_{\mathrm{Dirac}}$). The shift in the $V_{\mathrm{Dirac}}$ after the binding of IgG ($\Delta V_{\mathrm{Dirac}})$ can therefore be used for the quantification of IgG (Figure 3b).

### 3.2. Matrix Effect on the EGGFET Biosensors

#### 3.2.1. Matrix Effect on the $V_{\mathrm{Dirac}}$ of the EGGFET Biosensors

In EGGFET biosensors, the graphene channels are directly exposed to the electrolytes, which makes it important to learn if and how electrolytes would affect the operation of the EGGFET biosensors. The electrolyte matrices for bioanalysis are highly variant and complex; to better understand the underlying mechanisms of the matrix effects, we chose several simple electrolytes and studied the matrix effect as a function of their composition, pH and ionic strength. In this study, we focus on the matrix effect on the $V_{\mathrm{Dirac}}$ of the EGGFET which is directly related to its biosensing applications.

**Figure 4:**
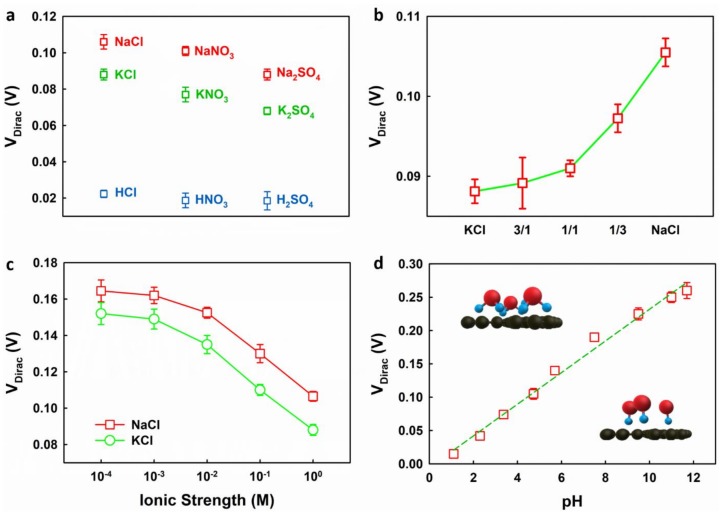
The Dirac voltage ($V_{\mathrm{Dirac}}$) of the EGGFET biosensors in (**a**) different electrolytes (all concentrations are 1 M except for Na_2_SO_4_, K_2_SO_4_ and H_2_SO_4_, which are 0.5 M); (**b**) mixture of KCl (1 M) and NaCl (1 M) with different mixing ratios (horizonal axis); (**c**) NaCl and KCl solution with different ionic strengths and (**d**) KCl solution (1 M) that was titrated with different pH. The schematics in (**d**) show the possible orientation of the H_3_O^+^ and OH^−^ on the graphene. The error bars indicate the standard deviation of the measured results of the five parallel channels.

We measured the transfer curves of a set of EGGFET biosensors in different electrolytes using a standard Ag/AgCl reference electrode as the gate and the $V_{\mathrm{Dirac}}$ was extracted from the transfer curves based on the linear regression analysis of $V_{g}$ vs. the slope of the transfer curves (see Supplemental Section 4, Figure S4). As shown in Figure 4a, the $V_{\mathrm{Dirac}}$ of the EGGFET biosensors exhibits a strong dependence on the composition of the electrolytes. In particular, the $V_{\mathrm{Dirac}}$ of the EGGFET in potassium salt electrolytes is lower than that in the corresponding sodium salt electrolytes of the same concentration. This ion-specific dependence was also reported in Heller’s report, which claimed that Li^+^ shows a stronger electrostatic gating effect than K^+^ [23]. Our result is in accordance with a simulation which took into account the polarization of both the graphene and the ions at the graphene–electrolyte interface, and suggested that K^+^ ions are more strongly absorbed onto graphene than Na^+^ ions [24]. Because both K^+^ and Na^+^ have one positive charge, the stronger adsorption of K^+^, which leads to a higher surface charge density on graphene, will introduce stronger n-doping in the graphene channel due to the electrostatic gating effect. The stronger n-doping effect of K^+^ compared with Na^+^ is further verified by the measurement of the $V_{\mathrm{Dirac}}$ of the EGGFET in the mixture of NaCl and KCl solutions with different ratios. As shown in Figure 4b, the $V_{\mathrm{Dirac}}$ shifts negatively as the ratio of KCl increases. In addition, the EGGFET exhibits different $V_{\mathrm{Dirac}}$ in NaCl, NaNO_3_ and Na_2_SO_4_ (all containing 1 M Na^+^), respectively, suggesting that the $V_{\mathrm{Dirac}}$ of EGGFET biosensors is also dependent on the type of anions they contain.

More significantly negative shift in $V_{\mathrm{Dirac}}$ was observed when acids were used as the electrolytes (Figure 4a). We further extended the pH range of the electrolyte by titrating HCl (0.1 M, in 1 M KCl) with KOH (0.1 M, in 1 M KCl). The titration allowed us to adjust the pH of the electrolytes while keeping the ionic strength relatively constant. The pH was monitored using a Hach pocket pro pH tester. A linear positive shift in $V_{\mathrm{Dirac}}$ with respect to the pH was observed, as shown in Figure 4d. This result is consistent with previous reports that proposed the application of EGGFET as pH sensors [7,25]. The pH dependence of $V_{\mathrm{Dirac}}$ suggests the strong adsorption of the hydronium and hydroxide ions on graphene as well as their n-doping and p-doping effects, respectively. In addition to the ion–π interaction which leads to the enhanced adsorption of H_3_O^+^ and OH^−^ on graphene, as discussed above, the dipole–π interaction might be a more important contributing factor [26]. Studies have indicated that polar molecules tend to orient in the direction that is normal to the graphene with strength sufficient to overcome thermal effects [27]. Due to the strong adsorption of these polar ions, the continuous transition from the n-doping H_3_O^+^ to p-doping OH^−^ causes the continuous positive shift of $V_{\mathrm{Dirac}}$ as the pH increases. Additionally, the transfer characteristics of the EGGFET were studied in electrolytes with different ionic strengths. NaCl and KCl solutions with different ionic strengths were prepared by successive 10-fold dilution of the stock solutions (1 M). The ionic strengths were calculated according to I=c(M) for monovalent salts; c(M) represents the molar concentration. As shown in Figure 4c, an increase in ionic strength causes a negative shift in $V_{\mathrm{Dirac}}$, which is in agreement with previous reports [23]. Because the overall doping effect of the electrolytes is determined by the competitive adsorption of the cations and the anions, this result indicates that Na^+^ and K^+^ surpass Cl^−^ in capability to dope graphene and reveals the significant impact of the ions in the electrolytes on the transfer characteristics of EGGFET biosensors. The minimum conductivity and the transconductance (the slope of the transfer curve) of EGGFET biosensors are not sensitive to the matrix effect (see Supplemental Section 5, Figure S5).

#### 3.2.2. Matrix Effect on the Potential of the Gate Electrode

**Figure 5:**
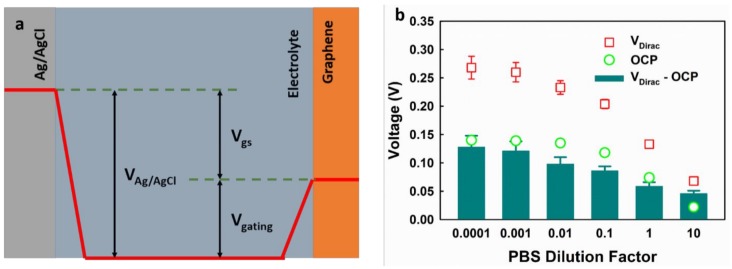
The impact of the gate electrode potential on EGGFET biosensors. (**a**) The potential distribution in EGGFET biosensors; (**b**) the $V_{\mathrm{Dirac}}$ of the EGGFET biosensors with the Ag/AgCl pseudo-reference electrode as the gate in different PBS diluents and the OCP between the Ag/AgCl pseudo-reference electrode and the standard Ag/AgCl reference electrode. The error bars indicate the standard deviations of the measurement results for the five parallel channels.

For the application in the EGGFET biosensors, a standard Ag/AgCl reference electrode is preferred due to its well-defined potential. However, standard Ag/AgCl reference electrodes are difficult to miniaturize and integrate with microfluidics devices. The Ag/AgCl pseudo-reference electrode is an alternative because its fabrication is compatible with microfabrication techniques [28]. In EGGFET biosensors, the real gate voltage that modulates the graphene channel is the voltage across the electrical double layer (EDL) [4], and the potential of the reference electrode must be considered in the determination of the $V_{\mathrm{Dirac}}$. The potential distribution in EGGFET biosensors with an Ag/AgCl reference electrode is shown in Figure 5a and can be described by Equation (1):

(1) $$V_{\mathrm{gating}}=V_{\mathrm{Ag}/\mathrm{AgCl}}-V_{\mathrm{gs}}$$

in which $V_{\mathrm{gating}}$ is the potential across the EDL, $V_{\mathrm{gs}}$ is the applied gate-to-source voltage, $V_{\mathrm{Ag}/\mathrm{AgCl}}$ is the potential of the Ag/AgCl electrode versus the electrolyte. In contrast to the standard Ag/AgCl reference electrode, the potential of the Ag/AgCl pseudo-reference electrode is not fixed but is determined by the composition of the electrolytes. In PBS, the potential of the Ag/AgCl pseudo-reference electrode can be estimated by the activity of Cl^−^ using the Nernst Equation (2):

(2) $$E_{\mathrm{Ag}/\mathrm{AgCl}}=E_{\mathrm{Ag}/\mathrm{AgCl}}^{o}-\frac{RT}{F}\ln\left(a_{{\mathrm{Cl}}^{-}}\right)$$

in which $E_{\mathrm{Ag}/\mathrm{AgCl}}^{0}$ is the standard potential, R is the universal gas constant, T is the temperature in Kelvins, F is Faraday’s constant and $a_{{\mathrm{Cl}}^{-}}$ is the activity of Cl^−^. We measured the $V_{\mathrm{Dirac}}$ of the EGGFET biosensors with an Ag/AgCl pseudo-reference electrode as the gate; we also measured the open circuit potential (OCP) of the Ag/AgCl pseudo-reference electrode with respect to the standard Ag/AgCl reference electrode in PBS with different dilution factors. As shown in Figure 5b, the OCP of the Ag/AgCl pseudo-reference electrode decreases as the concentration of PBS increases; comparatively, the $V_{\mathrm{Dirac}}$ also shifts downwards but with a larger amplitude, and the difference can be attributed to the matrix effects of the electrolyte on graphene, as discussed above.

#### 3.2.3. Matrix Effect on the Sensitivity of the EGGFET Biosensors

**Figure 6:**
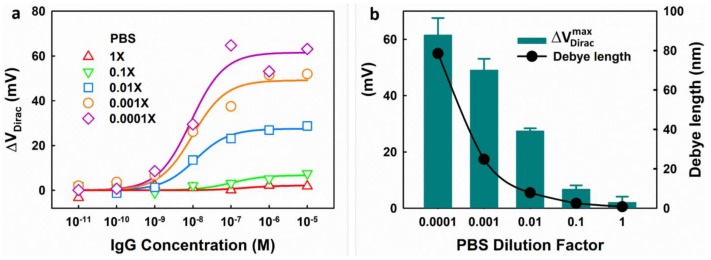
The impact of the ionic strength on the sensitivity of an EGGFET biosensor. (**a**) The response of the EGGFET biosensor to IgG under different diluents; (**b**) The maximum response ($\Delta V_{\;\mathrm{Dirac}}^{\max}$) of the EGGFET biosensor in different PBS diluents and the corresponding Debye length. The error bars indicate the standard errors of the estimates for the fitting.

In electrolytes, the electrostatic effect of charges is screened by the attraction of the opposite charges and the specific orientation of the dipoles around them. Debye length, which is dependent on the ionic strength of the electrolyte, is normally used to characterize the distance of the electrostatic effects’ persistence. The sensitivity of the FET biosensors is significantly limited by the Debye screening effect [29]. Figure 6a shows the responses of one EGGFET immunosensor to IgG in PBS with different dilution factors. In 1× PBS, no significant response was observed because the Debye length (around 0.7 nm) is smaller than the height of the functional layer (the linker + the receptor). As the concentration of PBS decreases, the charged molecules exhibit a stronger modulation capability due to the increase in Debye length and thus, a stronger response can be obtained. As shown in Figure 6b, the $\Delta V_{\;\mathrm{Dirac}}^{\max}$ under different ionic strengths are plotted in comparison with the corresponding Debye lengths which were calculated using Equation (3),

(3) $$\delta =\sqrt{\frac{\varepsilon kT}{2e^{2}N_{A}C}}$$

where, ε is the permittivity of the electrolyte, k is the Boltzman constant, T is the temperature in Kelvins, e is the elementary charge, $N_{A}$ is the Avogadro number and C is the concentration of the electrolytes in mol/m^3^. The results indicate that the Debye lengths are highly dependent on the ionic strength of the electrolytes, resulting in a strong impact on the sensitivity of the EGGFET biosensors.

As shown in Figure 6, higher sensitivity could be obtained by using PBS with lower concentrations; however, low concentrations also introduce significant uncertainty, possibly due to (1) the high resistance of the electrolytes of low concentrations which makes the voltage between the reference electrode and the graphene unstable; and (2) the fluctuation in the potential of the Ag/AgCl pseudo-reference electrode when the concentration of Cl^−^ is low, as discussed in Section 3.2.2. The results show that 0.01× PBS provides the best performance, which compromises the sensitivity and measurement uncertainty.

### 3.3. EGGFET Immunoassay

#### 3.3.1. Response of the EGGFET Immunosensor to IgG

As discussed above, matrix effects including the composition, ionic strength and pH of the electrolytes can significantly impact the operation and response of a EGGFET biosensor. These matrix effects must be carefully regulated during applications of EGGFET biosensors, otherwise high uncertainty or even false results might be obtained. Inspired by the well-developed enzyme-linked immunosorbent assay (ELISA), a novel EGGFET biosensor immunoassay (Figure 1a) was developed to allow good regulation of the matrix effects; this has the potential to improve its biosensing performance for practical applications.

**Figure 7:**
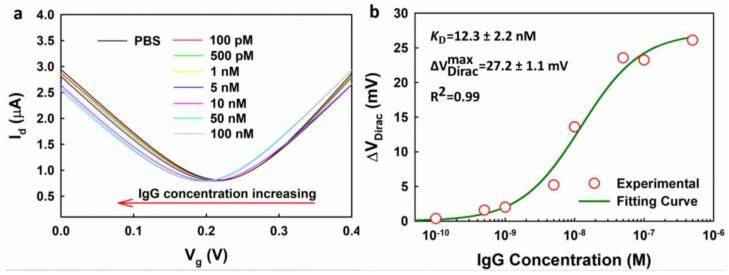
The responses of an EGGFET immunosensor to IgG. (**a**) The continuous shifts of the transfer curves of the EGGEFT immunosensor upon the addition of IgG with different concentrations dissolved in 0.01× PBS. The source to drain voltage was set to be 0.01 V; (**b**) $\Delta V_{\mathrm{Dirac}}$ with respect to the concentrations of IgG. The uncertainties of the fitting parameters indicate the standard errors of the estimates for the fitting.

We first measured the response of one EGGFET immunosensor to IgG. It is worth noting that 0.01× PBS was used as the detection buffer because it provides relatively high sensitivity and low uncertainty, as discussed earlier, and is shown in Figure 6. The typical transfer curves of the EGGFET immunosensor after incubation with IgG of different concentrations are shown in Figure 7a. Continuous negative shifts of the transfer curves were observed due to the positive charges of the IgG molecules which introduce n-type doping and positive shift of the Fermi level in graphene. The responses of the EGGFET immunosensor are plotted with respect to the IgG concentrations, as shown in Figure 7b. The plots were fitted with the Hill–Langmuir equation (Equation (4)):

(4) $$\Delta V_{\mathrm{Dirac}}=\Delta V_{\mathrm{Dirac}}^{\max}\times \frac{\left[\mathrm{IgG}\right]}{\left[\mathrm{IgG}\right]+K_{D}}$$

where, $K_{D}$ is the dissociation constant of the aptamer-IgG complex. The fitting indicates that there is a linear relationship between $\Delta V_{\mathrm{Dirac}}$ and the occupation ratio of the binding sites on graphene. The fitting yields $K_{D}$ of 12.3 nM which is higher than the value provided by the manufacturer (8.4 nM), and this can be attributed to the suppressed on-rate caused by the spatial orientation of the aptamer.

**Figure 8:**
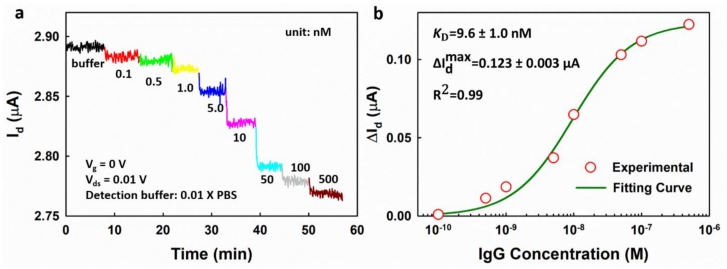
The real-time measurement of IgG using the EGGFET immunosensor. (**a**) The change in $I_{d}$ upon successive addition of IgG with increasing concentrations from 0.1 nM to 500 nM; (**b**) drain current ($\Delta I_{d}$ ) with respect to the concentrations of IgG. The uncertainties of the fitting parameters indicate the standard errors of the estimates for the fitting.

The EGGFET biosensor was also used for real-time measurement by monitoring the drain current ($I_{d}$) at a fixed gate voltage. The drain current of the EGGFET immunosensor at $V_{g}$ = 0 V was monitored upon successive addition of IgG with increasing concentrations. $V_{g}$ at 0 V was chosen because the transconductance is relatively high at $V_{g}$ = 0 V, as shown in Supplemental Section 3 Figure S3c, in which higher transconductance showing higher sensitivity. As shown in Figure 8a, the drain current decreases as the IgG concentration increases, which is consistent with the negative shifts of the transfer curves shown in Figure 7a. The drain current changes can also be well fitted with the Hill–Langmuir equation as a function of the IgG concentration, and a dissociation constant $K_{D}$ of 9.6 nM was obtained (Figure 8b).

**Figure 9:**
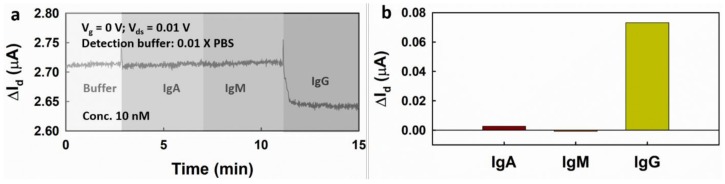
The selectivity of the EGGFET immunosensor for IgG detection. (**a**) Responses of the EGGFET immunosensor to the successive addition of IgA, IgM and IgG; (**b**) $\Delta I_{d}$ of the EGGFET immunosensor as responses to IgA, IgM and IgG.

To test the selectivity of the EGGFET immunosensor, immunoglobulin A (IgA) and immunoglobulin M (IgM) and IgG were added successively into the channel and the drain current was monitored. No significant drain current change was observed upon the additions of IgA and IgM, which indicates that there is a good selectivity of the EGGFET immunosensor to IgG (Figure 9a,b).

#### 3.3.2. Standard Operation Protocol for the EGGFET Immunoassay

**Figure 10:**
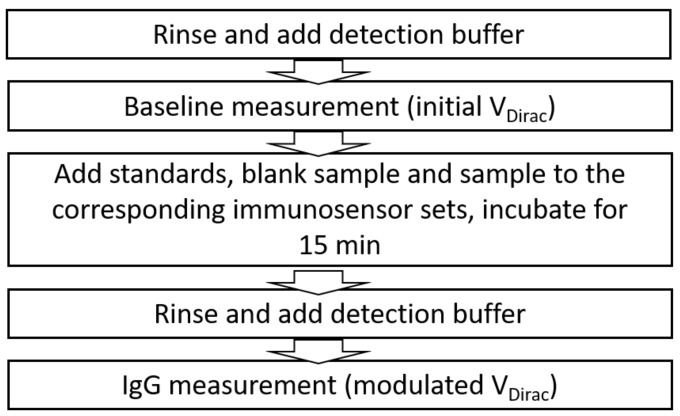
Standard operation protocol for the EGGFET immunoassay for IgG measurement.

To allow the regulation of the matrix effect and obtain reliable test results, we developed a standard operation protocol for the EGGFET immunoassay, as shown in Figure 10. For a better understanding of the operation of the EGGFET immunoassay, several terms used in this paper are clarified as follows: (1) the detection buffer is the electrolyte in which the transfer curves of the immunosensors are measured; and (2) the washing buffer is the solution used to rinse the channel and remove the non-specifically adsorbed biomolecules. In this work, 0.01× phosphate buffer saline (PBS) was used as the detection buffer and washing buffer during the experiment, unless otherwise specified.

The operation protocol of the EGGFET immunoassay is described as follows: (1) The seven immunosensor sets were filled with detection buffer after rinsing with washing buffer three times; (2) the transfer curves of the EGGFET immunosensors were measured and the initial $V_{\mathrm{Dirac}}$ was extracted; (3) the EGGFET immunosensors were incubated with standards, blank solution and sample for 30 min, according to the function assignment shown in Figure 1a; (4) the immunosensors were rinsed with washing buffer three times and then filled with detection buffer; (5) the transfer curves of the EGGFET immunosensors were measured and the modulated $V_{\mathrm{Dirac}}$ was extracted). We hereby emphasize the $V_{\mathrm{Dirac}}$ is measured in the detection buffers instead of the original samples to minimize or exclude the effects caused by the sample matrices.

#### 3.3.3. Spike-and-Recovery Test

**Figure 11:**
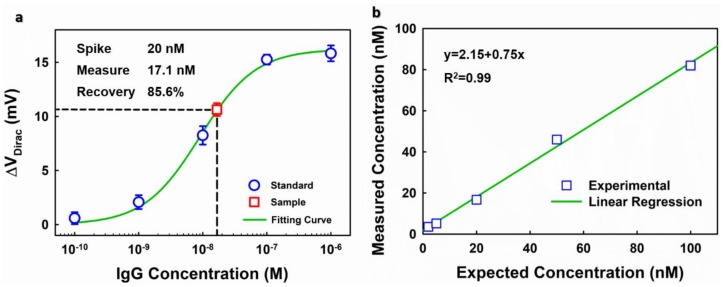
Evaluation of the EGGFET immunoassay. (**a**) The spike-and-recovery test of the EGGFET immunoassay for IgG detection. The error bars refer to the standard deviations of the measurement results for the five parallel channels; (**b**) The linearity-of-dilution assessment of the EGGFET immunoassay.

A spike-and-recovery test was used for examining the performance of the EGGFET immunoassay. The standards were prepared by dissolving human IgG (essentially salt-free, lyophilized powder, Sigma Aldrich) in 1× PBS to specific concentrations; goat serum (Sigma Aldrich) was used as the blank sample for the negative control. The samples to be measured were prepared by spiking goat serum with human IgG with specific concentrations. Following the protocol mentioned above, the initial and modulated $V_{\mathrm{Dirac}}$ of EGGFET immunosensors, before and after IgG binding, were measured and $\Delta V_{\mathrm{Dirac}}$ was calculated. A representative result is shown in Figure 11a in which samples spiked with 20 nM IgG was measured. The calibration curve was obtained by fitting the responses to the standards using the Hill–Langmuir equation, and the IgG concentration of the sample was calculated based on the calibration curve. Figure S6 shows the spike-and-recovery test results of the samples that are spikes with IgG of 2 nM, 5 nM, 50 nM and 100 nM, respectively (Supplemental Section 6). The results suggest that the devices that were fabricated on the same chip exhibit high uniformity in performance. However, there was a significant variability among different chips, for example, the maximum Dirac voltage shift ($\Delta V_{\;\mathrm{Dirac}}^{\max}$) varied from 15 mV to 25 mV. This significant “chip-to-chip” variation is one of the main challenges that hinders the application of graphene-based biosensors. Our immunoassay takes advantage of the uniformity of the devices in a single chip and integrates the self-calibration capability, suggesting great potential for practical applications. The detection range is estimated to be around 2~50 nM with a coefficient of variation (CV) of less than 20% (see Supplemental Section 7, Figure S7).

The reliability of the EGGFET immunoassay was further verified by a linearity-of-dilution assessment. As shown in Figure 11b, the measured concentrations were plotted with respect to the spike concentrations. The good linearity indicates that the matrix effects have been well regulated. Reproducible results can be achieved with an 85–95% reproducible recovery rate, suggesting the feasibility of the EGGFET immunoassay for IgG measurement. The discrepancy between the measured results and the actual concentrations might be attributed to the constituent difference between standards and samples and can be reduced by optimizing the composition of the standard’s matrix.

In summary, the EGGFET immunoassay exhibits high reliability due to (1) the high uniformity in the electrical properties (particularly the $V_{\mathrm{Dirac}}$) of the sensing channels in a single chip (see Supplemental Section 8, Figure S8); (2) the good regulation of the matrix effects and the operation procedure; (3) the statistical validation of the measured results using multiple parallel EGGFET immunosensors. The performance can be further improved by optimizing the operation parameters, such as the composition/pH of the detection buffer; this is beyond the scope of this paper and we will report the results separately. This assay can also be used for detecting other biomolecules by functionalizing graphene channels with their corresponding bioreceptors. It is worth noting that the operation parameters should be optimized depending on the physical and physiological properties of the target biomolecules, e.g., the pH of the buffer should be adjusted based on the isoelectric point (pI) of the target proteins. Compared with traditional immunoassay techniques, such as the commercial ELISA, the EGGFET immunoassay is label-free and easy to use. It does not rely on specific signal collecting equipment and can be easily integrated into electrical measurement and sample delivery systems, suggesting its great prospect for point-of-care applications.

## 4. Conclusions

In this paper, matrix effects of electrolytes were investigated for electrolyte-gated graphene biosensors; the results show that the composition, pH, and ionic strength of the electrolyte used have considerable impact on the characteristics and performance of EGGFET due to polarization-induced interaction at the interface between electrolyte and graphene. The study on the effect of the matrix on the EGGFET biosensors provides a more in-depth understanding of the characteristics, optimization, and application of the EGGFET biosensors. An EGGFET immunoassay was shown to allow good regulation of the matrix effects and statistical validation of the measured results in human IgG detection, suggesting its great potential for point-of-care applications.

## Supplementary Materials

The following are available online at http://www.mdpi.com/2072-666X/9/4/142/s1, Figure S1: Ag/AgCl electroplating setup, Figure S2: Characterization of graphene upon functionalization, Figure S3: Optimization of the operation parameters of the EGGFET biosensor, Figure S4: Derivation of the $V_{\mathrm{Dirac}}$ from the transfer curves, Figure S5: The transfer curves of one representative EGGFET in KCl solutions of different concentrations., Figure S6: The spike-and-recovery test for samples that are spiked with IgG, Figure S7: Determination of the detection range of the EGGFET biosensor for IgG detection, Figure S8: The study on the device uniformity.
