# Supplementary material for: Matrix Effect Study and Immunoassay Detection Using Electrolyte-Gated Graphene Biosensor
Source: Micromachines (Basel). 2018 Mar 23;9(4):142. doi: 10.3390/mi9040142 (PMC6187333; doi:10.3390/mi9040142)
Supplement: Supplementary file 1 [file micromachines-09-00142-s001.pdf]

Supplementary Materials: Matrix Effect Study and Immunoassay Detection Using Electrolyte-Gated Graphene Biosensor

Jianbo Sun and Yuxin Liu

1. The Electroplating of the Ag/AgCl Pseudo-Reference Electrode

The schematic and experimental setup for the fabrication of the Ag/AgCl pseudo-reference electrode are shown in Figure S1.

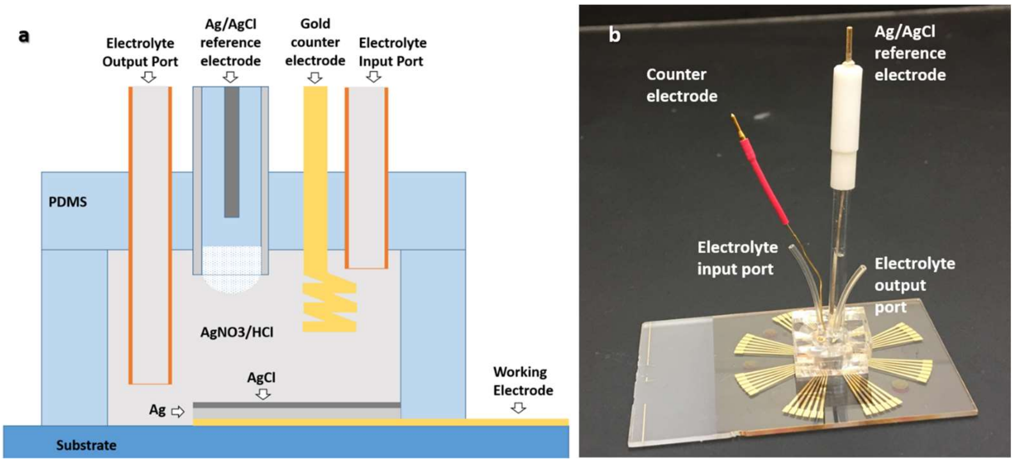

Figure S1. Ag/AgCl electroplating setup. (**a**) Schematic of the electroplating chamber. (**b**) A picture of the electroplating setup.

2. Characterization of the Functionalization of Graphene

X-ray photoelectron spectroscopy (XPS) and Raman spectroscopy were used for the characterization of the functionalization of graphene. The samples for characterization are glass slides with transferred CVD graphene on them and the characterizations were performed after each functionalization step. As shown in Figure S2a, the bare graphene exhibits a single C1s peak which indicates that cleaning graphene was obtained. After the immobilization of 1-pyrenebutyric acid *N*-hydroxysuccinimide ester (PBASE), new C1s peak at 289.5 eV and N1s peak at 402 eV arise which are attributed to the O-N-C=O in the *N*-Hydroxysuccinimide (NHS) group in the PBASE [1]. After conjugation with aptamer, strong C1s peaks at 288 eV and 286.5 eV and N1s peak at 400.5 eV arise which are common in DNA [2]. These peaks are intensified after blocking with bovine serum albumin (BSA) which are typically for protein functional groups [3].

A standard Raman spectrum was obtained after graphene transfer (Figure S2b). After PBASE immobilization, new peak arises just by the G peak on the right side which is assignable to the resonance introduced by the pyrene-graphene stacking [4]. The enhancement of the D peak at 1380 cm^{-1} is due to the disorder arising from π-π orbital hybridization [4]. After conjugation with aptamer, the two peaks are further intensified and a broad band appears at around 2900 cm^{-1} which is also reported by [5]. But the assignment of these peaks is still to be studied. Normally the broad band at around 2900 cm^{-1} is assigned to the aliphatic C-H stretching.

The transfer characteristics of the graphene field effect transistor (GFET) were measured after each functionalization process. As shown in Figure S2c, significant positive shift was observed after functionalization and blocking due to the p-doing effect of pyrene group [6] and negatively charged BSA [7]. After incubating with 1μM IgG, the transfer curve was shifted negatively which suggests the positive charge of IgG and its n-doping effect on graphene.

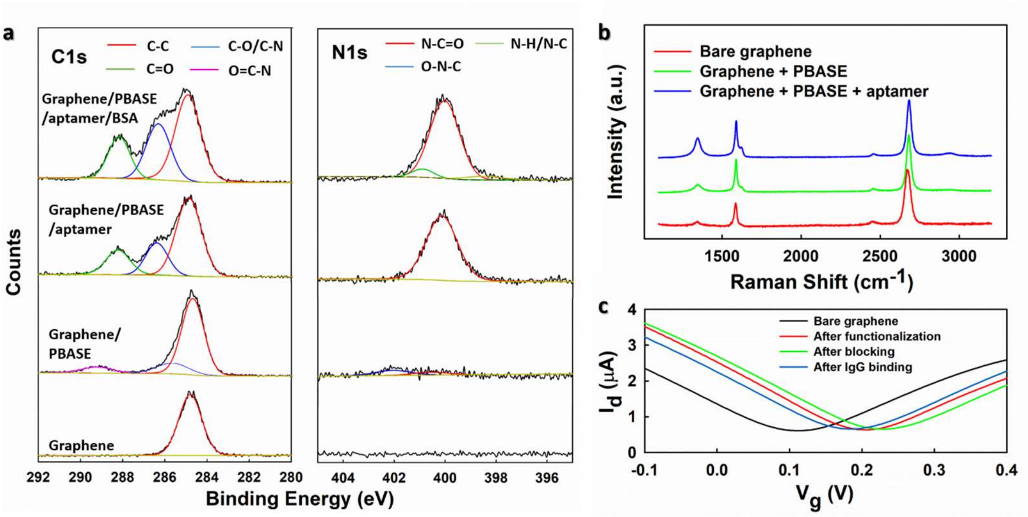

Figure S2. Characterization of graphene upon functionalization. (**a**) C1s and N1s X-ray photoelectron spectroscopy (XPS) spectrum. (**b**) Raman spectrum. (**c**) Transfer curves measured in 0.01× PBS (Base Pair Biotechnologies, Pearland, TX, USA) with V_{ds}= 0.01 V.

3. Optimization of the Operation Parameters

The hysteresis of the transfer curves of GFET upon reversal of the sweeping direction has been previously reported [8]. Our experiments indicate that the hysteresis also happens in EGGFET and have a direct impact on the detection precision for EGGFET based biosensors (Figure S3a). The hysteresis shows great dependence on the gate voltage sweeping rate and range. As shown in Figure S3b, the hysteresis can be effectively reduced at scan rate lower than 10 mV/s. All the transfer curves were obtained at gate voltage sweep rate of 10 mV/s unless otherwise specified. We propose the hysteresis is caused by the lagging of the ions movement in response to the gate voltage change.

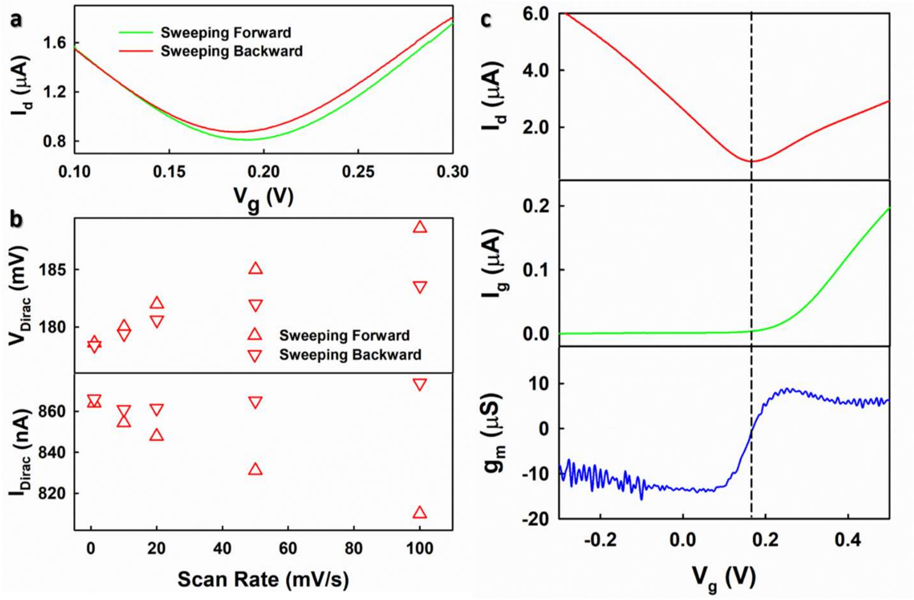

Figure S3. Optimization of the operation parameters of the electrolyte-gated graphene field effect transistor (EGGFET) biosensor. (**a**) Hysteresis of the forward and backward transfer curves measured with scan rate of 100 mV/s. (**b**) The V_{Dirac} and I_{Dirac} (minimum drain current) of the forward and backward transfer curves at different scan rates. (**c**) The transfer curve (I_d), leakage current (I_g) and transconductance (g_m) of the EGGFET measured in 0.01× PBS.

To maximize the modulation capability of the V_g, V_{ds} was set to be 0.01 V which is smaller relative to the V_g sweeping range and yields I_d of several microamperes. The leakage current (I_g) was measured and leakage current was observed at V_g > +0.2V (in 0.01X PBS with standard Ag/AgCl reference electrode) which is attributed to the electrochemical reduction of water (Figure S3c). To avoid the change of the electrolyte composition caused by water reduction, the V_g sweeping range should be kept away from the water reduction potential. Our devices work well in the range of V_g < +0.5 V; as shown in Figure S3c, even though the leakage current might cause some distortion of the transfer curves at high V_g, it won't have significant impact on the measurement of the V_{Dirac}. One of the key parameters characterizing the sensitivity of the field effect transistor (FET) biosensors is the transconductance (g_m). As shown in Figure S3c, the maximum g_m was obtained at around 0.1 V and 0.3 V. V_g of 0 V was adopted for the continuous measurement of I_d for the detection of IgG because the sensitivity of the biosensor is the highest near 0 V (maximum g_m) and the biosensor gives the most stable response in this range.

4. Determination of the Dirac Voltage

The determination of the V_{Dirac} from the transfer curves was conducted using Excel. The original transfer curve is shown in Figure S4a. The slope of I_d vs V_g was calculated using the SLOPE function (Figure S4b). The slope function was calculated over 7 data points to reduce noise. Since the slope goes thru zero at the Dirac voltage, we plotted the V_g vs the slope for the data near the zero crossing and then fitted the small piece of data (20 points) with a straight line using the LINEST function (Figure S4c). The LINEST function provides the slope, intercept and their standard deviations. The intercept is the Dirac voltage and the standard deviation of the intercept is the uncertainty in the Dirac voltage.

The linear regression analysis allows more precise determination of the Dirac voltage with an uncertainty of less than 0.2 mV. The direct determination of the Dirac voltage by locating the minimum I_d generates uncertainty of several millivolt due to the measurement (as shown in Figure S4d). In the example as demonstrated in Figure S4, the Dirac voltage determined by linear regression of the V_g vs the slope is +0.21856 V with an uncertainty of 0.00017 V, while the V_g with the minimum I_d is +0.220 V.

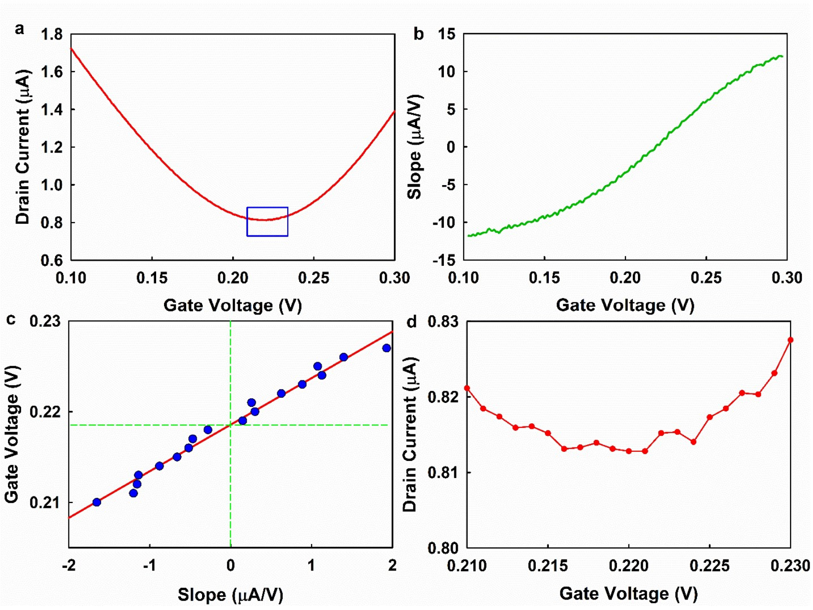

Figure S4. Derivation of the V_{Dirac} from the transfer curves. (**a**) The original transfer curve (I_d vs V_g). (**b**) The slope of I_d vs V_g. (**c**) V_g vs slope and the linear regression near. (**d**) Enlarge view of the transfer cure as indicated by the box in a).

5. The Matrix Effect on the Minimum Conductivity and Transconductance of the EGGFET

In Figure S5, the transfer curves of the EGGFET in KCl solution of different concentrations are shown which corresponds to Figure 3c in the main text. Continuous negative shifts of the transfer curves are observed; however, the minimum conductance and the slope of the transfer curves are almost unchanged. It suggests that the minimum conductance and the sensitivity (the slope of the transfer curve in linear region) are not sensitive to the matrix effect, which is consistent with previous reports [9].

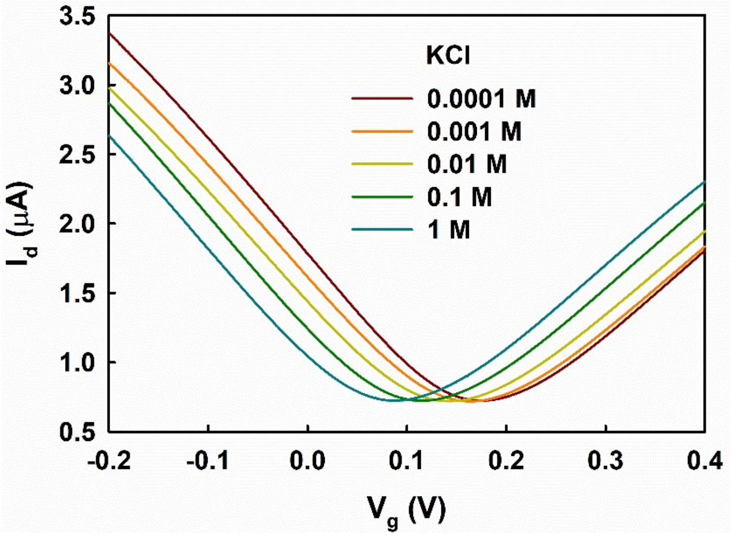

Figure S5. The transfer curves of one representative EGGFET in KCl solutions of different concentrations.

6. Spike-and-Recovery Test

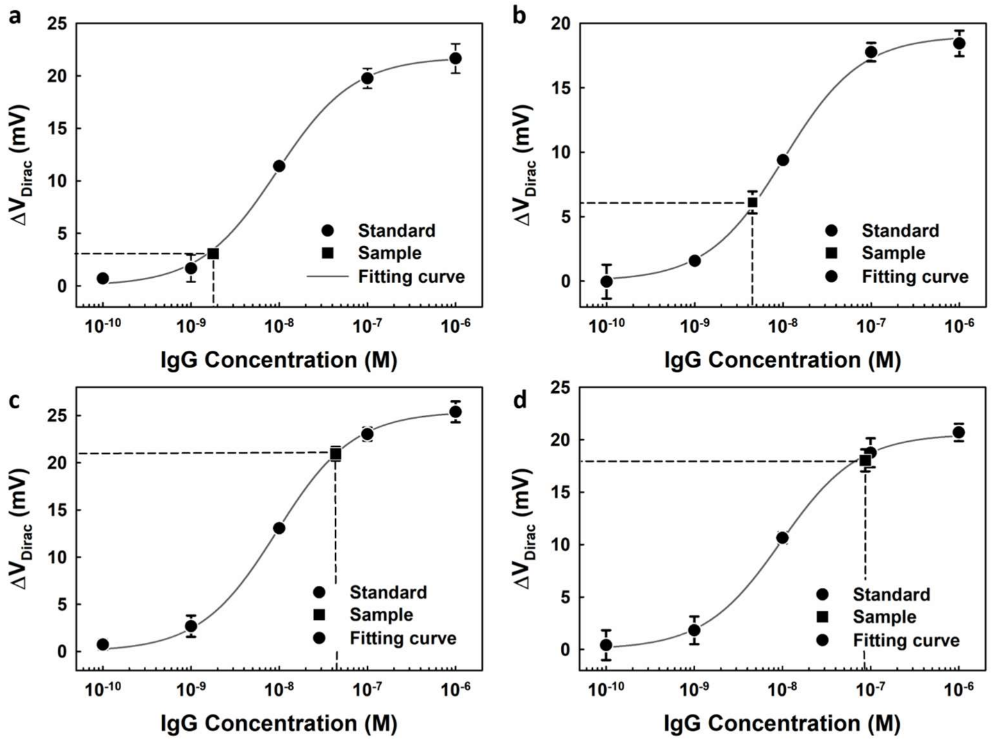

Figure S6. The spike-and-recovery test for samples that are spiked with IgG of (**a**) 2 nM, (**b**) 5 nM, (**c**) 50 nM and (**d**) 100 nM. The errors bars indicate the standard deviations of the measured results of the five parallel channel is each set.

7. Determination of the Detection Range

The detection range is the concentration range that can be reliably measured by the biosensor. Here we determine the detection range of the EGGFET biosensors based on the relative standard deviation (RSD) of the concentration estimates. Firstly, the response is fitted using the Hill-Langmuir equation as shown in Figure S7a. The slope of the fitting curve is derived as shown in Figure S7b. The standard deviation (SD) of the concentration estimated (Figure S7c) is calculated by dividing the SD of measurement by the slope. The RSD is the ratio of the SD to the estimated concentration and plotted in Figure S7d. For bioanalysis, such as enzyme-linked immunosorbent assay (ELISA), a coefficient of variance (CV) of 20% is empirically accepted. Therefore, the detection range of the EGGFET biosensor is determined to be around 2–50 nM which corresponds to RSD < 20% as shown in Figure S7d. This method determines the detection range based on the concentration estimates instead of the measurement reading, generates results with certain precision. It is applicable for bioanalysis that is regulated by nonlinear equation.

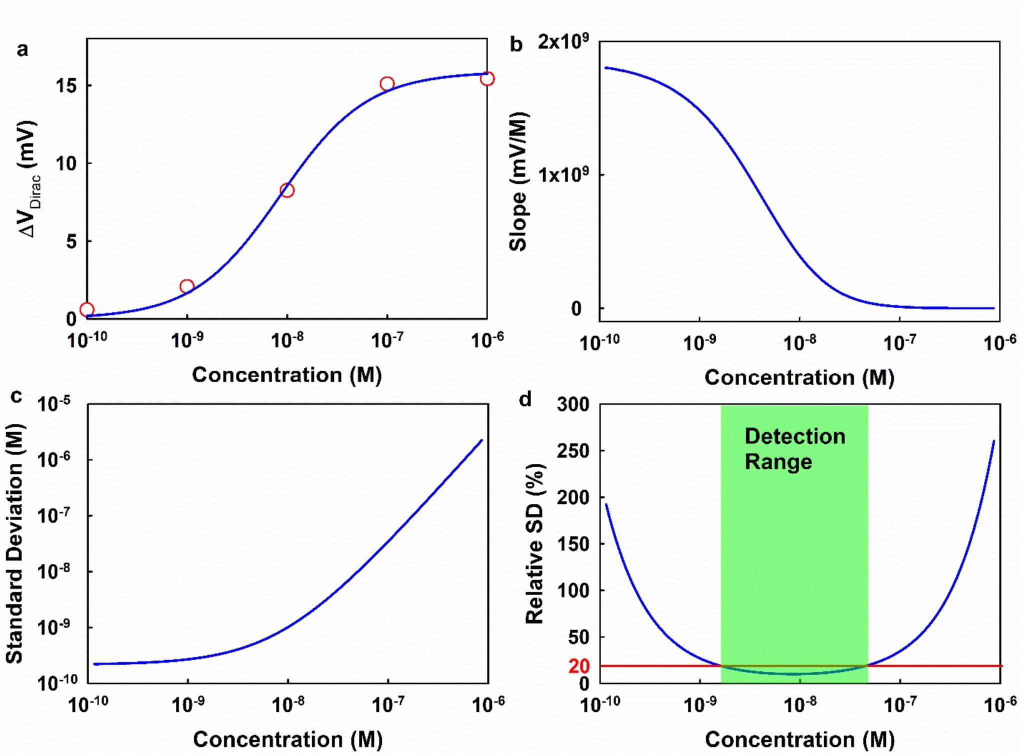

Figure S7. Determination of the detection range of the EGGFET biosensor for IgG detection. (**a**) The fitting curve of the measurement results; (**b**) The slope of the fitting curve; (**c**) The SD of the concentration estimates; (**d**) the RSD of the concentration estimated with respect to the concentration.

8. Uniformity of the Electrical Properties of the Graphene Channels

The transfer curves of all the 35 graphene channels in a representative device along with the analysis of the minimum conductance and Dirac point are shown in Figure S8. All the channels exhibit the ambipolar behavior (Figure S8a). The minimum conductance ranges from 30 to 80 μS with a large deviation (Figure S8b), while the Dirac points exhibit relatively small deviation (Figure S8c). We attribute these dispersions to the imperfections in the graphene which are inevitable with the currently existing techniques, e.g. defects and impurities introduced during the synthesis of graphene and the device fabrication processes. The conductance of the graphene channels are highly subject to these imperfections which leads to the large deviation. However, because the operation of our device is based on the measurement of the Dirac point, the deviation in the conductance has no significant impact on the measurement results. The Dirac point is relatively stable in our devices which could be attributed to the deep cleaning of graphene using the electrolytic method [10].

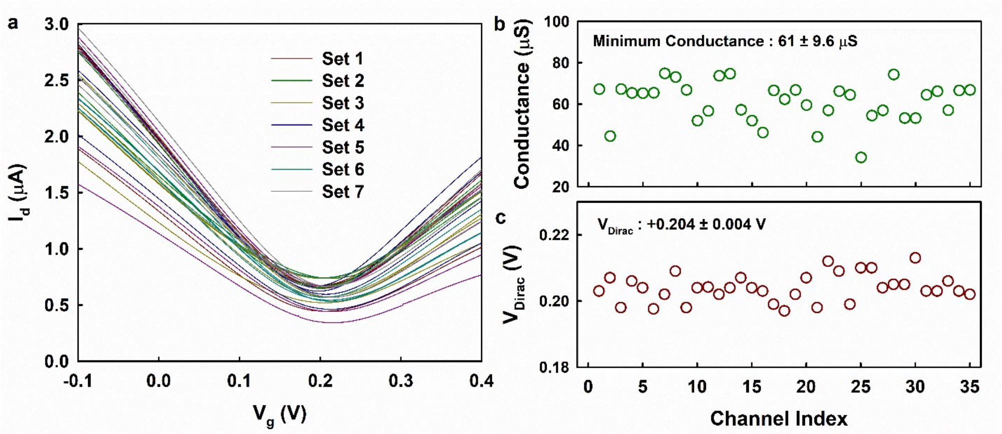

Figure S8. The study on the device uniformity. (**a**) The transfer curves of the all the 35 channels in a representative immunoassay device (7 sets with 5 channels in each of them); (**b**) the distribution of the minimum conductance and (**c**) the distribution of the Dirac voltage. The uncertainties indicate the standard deviations of the distribution.

Reference

1. Yang, M.; Teeuwen, R. L. M.; Giesbers, M.; Baggerman, J.; Arafat, A.; de Wolf, F. A.; van Hest, J. C. M.; Zuilhof, H. One-step photochemical attachment of NHS-terminated monolayers onto silicon surfaces and subsequent functionalization. *Langmuir* **2008**, *24*, 7931–7938, doi:10.1021/la800462u.
2. Ptasińska, S.; Stypczyńska, A.; Nixon, T.; Mason, N. J.; Klyachko, D. V.; Sanche, L. X-ray induced damage in DNA monitored by X-ray photoelectron spectroscopy. *J. Chem. Phys.* **2008**, *129*, 065102, doi:10.1063/1.2961027.
3. Roguska, A.; Pisarek, M.; Andrzejczuk, M.; Dolata, M.; Lewandowska, M.; Janik-Czachor, M. Characterization of a calcium phosphate–TiO2 nanotube composite layer for biomedical applications. *Mater. Sci. Eng. C* **2011**, *31*, 906–914, doi:10.1016/j.msec.2011.02.009.
4. Liu, Y.; Yuan, L.; Yang, M.; Zheng, Y.; Li, L.; Gao, L.; Nerngchamnong, N.; Nai, C. T.; Sangeeth, C. S. S.; Feng, Y. P.; Nijhuis, C. A.; Loh, K. P. Giant enhancement in vertical conductivity of stacked CVD graphene sheets by self-assembled molecular layers. *Nat. Commun.* **2014**, *5*, 5461, doi:10.1038/ncomms6461.
5. Joseph, D.; Seo, S.; Williams, D. R.; Geckeler, K. E. Double-stranded DNA-graphene hybrid: preparation and anti-proliferative activity. *ACS Appl. Mater. Interfaces* **2014**, *6*, 3347–3356, doi:10.1021/am405378x.
6. Dong, X.; Fu, D.; Fang, W.; Shi, Y.; Chen, P.; Li, L.-J. Doping single-layer graphene with aromatic molecules. *Small* **2009**, *5*, 1422–1426, doi:10.1002/smll.200801711.
7. Böhme, U.; Scheler, U. Effective charge of bovine serum albumin determined by electrophoresis NMR. *Chem. Phys. Lett.* **2007**, *435*, 342–345, doi:10.1016/j.cplett.2006.12.068.
8. Wang, H.; Wu, Y.; Cong, C.; Shang, J.; Yu, T. Hysteresis of electronic transport in graphene transistors. *ACS Nano* **2010**, *4*, 7221–7228, doi:10.1021/nn101950n.
9. Heller, I.; Chatoor, S.; Männik, J.; Zevenbergen, M. A. G.; Dekker, C.; Lemay, S. G. Influence of electrolyte composition on liquid-gated carbon nanotube and graphene transistors. *J. Am. Chem. Soc.* **2010**, *132*, 17149–17156, doi:10.1021/ja104850n.
10. Sun, J.; Finklea, H. O.; Liu, Y. Characterization and electrolytic cleaning of poly(methyl methacrylate) residues on transferred chemical vapor deposited graphene. *Nanotechnology* **2017**, *28*, 125703, doi:10.1088/1361-6528/aa5e55.
